# Use of connected injection device has a positive effect on catch-up growth in patients with growth disorders treated with growth hormone therapy

**DOI:** 10.3389/fendo.2024.1450573

**Published:** 2024-10-04

**Authors:** Antonio de Arriba, Paula van Dommelen, Martin O. Savage

**Affiliations:** ^1^ Pediatric Endocrinology Unit, Hospital Miguel Servet, Zaragoza University, Zaragoza, Spain; ^2^ Department of Child Health, The Netherlands Organization for Applied Scientific Research (TNO), Leiden, Netherlands; ^3^ Centre for Endocrinology, William Harvey Research Institute, Barts and the London School of Medicine & Dentistry, Queen Mary, University of London, London, United Kingdom

**Keywords:** growth hormone, adherence, growth response, height gain, digital tool

## Abstract

**Introduction:**

Human growth hormone (hGH) therapy in children can be administered by subcutaneous injection using either a manual non-connected device, which is a portable injection pen loaded with a pre-filled cartridge, or an electronic connected device. The electronic device is connected to a platform where adherence data is recorded and available for health care professionals (HCPs) and patient support programs. Real-world data used in the clinic, includes regular monitoring of adherence data which are shared with families during patients’ visits and aim to determine the root causes of poor adherence. This study aimed to identify whether there are differences in growth during the first four years of treatment depending on the device, i.e. non-connected versus connected devices.

**Methods:**

This retrospective study reports treatment of either GH deficiency or short stature secondary to birth size small for gestational age (SGA) in 174 pediatric patients attending Miguel Servet Hospital, Zaragoza, Spain. hGH treatment was administered with manual non-connected devices in 87 patients and 87 patients used connected devices. Height was followed for 4 years after start of hGH therapy.

**Results:**

In total, 57% of subjects had GHD and 43% were SGA. Height standard deviation score (HSDS) at treatment start was higher (p<0.001) in the non-connected device group compared to the connected device group. Change of HSDS in the connected device group was significantly higher in the second (+0.13), third (+0.20) and fourth (+0.23) year of treatment compared to the non-connected group after adjustment for age and HSDS at treatment start, sex, indication, dose and Tanner stages during treatment, and timing of measurements.

**Discussion:**

These results support the use of the connected device for hGH treatment of pediatric growth disorders.

## 1 Introduction

Two of the commonest pediatric disorders for which growth hormone (GH) therapy is licensed by the European Medicines Agency are GH deficiency (GHD) and short stature related to birth size small for gestational age (SGA) (1). GHD is characterized by the inadequate secretion of GH from the anterior pituitary gland and is treated by replacing GH with regular injections of synthetic recombinant human GH (hGH), produced by genetically engineered bacteria, manufactured by recombinant DNA technology. Children with GH deficiency need regular follow up so that the dose of hGH can be adjusted as they increase height and weight (2). They also need regular monitoring, not only for the effects of treatment but for any side effects that might occur. The growth response to GH therapy is influenced by several factors which include, the initial diagnosis, the age of the patient and the severity of the GH deficiency, adherence to treatment as well as individual responsiveness (3).

Being born SGA is defined as having a birth weight and/or birth length below −2 standard deviation score (SDS) for gestational age (4). Early identification and referral of children with persistent short stature after SGA birth is very important. hGH treatment can be initiated from an age of 4 years in Europe (1).

There are different devices for the subcutaneous administration of hGH safely and effectively. These devices can be classified into two main categories: manual devices and electronic devices. Manual devices (non-connected) consist of the injection pen, which is portable and easy to use for precise subcutaneous administration. Pre-filled cartridges are loaded into the pen which allows dose-adjustment, using a dial.

Electronic devices (connected) are similar to injection pens but feature digital characteristics that allow for greater precision in dosing and hormone administration. Typically, these devices have functions such as dose-memory, automatic dose adjustment, and administration logs.

The main differences between manual and electronic devices are the opportunities for healthcare professionals (HCPs) to act on adherence data and give support to identify the roots of poor adherence which will contribute to overall response to therapy. Some electronic devices come with additional features such as dose-memory, automatic dose adjustment, and administration logs, which can make the process more convenient and safe. Some people may find electronic devices easier to use due to the automated features they offer, while others may prefer the simplicity of manual devices.

It is important to note that the choice between an electronic and a manual device will depend on the individual needs and preferences of the patient, the doctor’s recommendation, and the availability of devices in the market. It is always crucial to follow the doctor’s instructions and receive proper training in the use of the selected device.

Measuring adherence to hGH therapy is generally difficult and mostly based on patient self-reported questionnaires, prescription records, or vial counting. It is not possible to objectively monitor GH treatment adherence with pen administration devices; any measure being reliant on diary cards and interviews with patients or their parents. eHealth-based ecosystems with automatic adherence recording and data transmissions allow proactive close monitoring of adherence and provide targeted support for individuals and groups of patients (5).

This retrospective analysis of real-world data from patients with growth disorders attending a single pediatric endocrinology clinic aimed to identify whether there are differences in growth during the first four years of treatment depending on the device, i.e. non-connected versus connected devices. The primary objective was to determine whether there were any differences in growth four years after treatment start according to the device in patients with GHD and SGA. The secondary objectives were to determine whether there were any differences in growth at one, two and three years after treatment initiation according to the device in patients with GHD and SGA.

## 2 Materials and methods

### 2.1 Study population

This is a longitudinal, retrospective and observational study in patients followed up in the pediatric endocrinology clinic of the tertiary care Miguel Servet Hospital, Zaragoza, Spain who underwent treatment for idiopathic GHD or short stature related to birth size SGA. In children with auxological data suggestive of GHD, GHD was defined as peak GH concentration <7 µg/L during two provocation tests, clonidine and insulin-induced hypoglycemia. The definition of short stature related to birth size SGA was height SDS <-2.5 at or after 4 yours of age and associated with birth weight and or length <-2 SDS (6). Both conditions are approved by the EMA for treatment with recombinant hGH.

### 2.2 Inclusion criteria

Patients on treatment with hGH with a diagnosis of idiopathic GHD or short stature related to birth size SGA with treatment approved by the regional Growth Hormone Committee;

- At least 4 years of uninterrupted hGH treatment;
- Age under 18 years old at start of hGH therapy;
- Signed informed consent.

We excluded patients when it was impossible to collect their data or if there was a change of device (from non-connected to connected or vice versa) during follow-up, if they had any indication other than GHD or SGA, when there was organic etiology or comorbidities that could affect growth and height outcomes (cancer, congenital heart disease, cerebral palsy, chromosome disorders or genetic syndromes), or patients that received concomitant treatment with luteinizing hormone-releasing hormone analogues.

### 2.3 Choice of device

Patients were started on the injection device which was chosen by the family and patient from a selection of options, making the decision together with their doctor.

### 2.4 Connected injection device

The connected electronic device is connected to a platform where adherence data is available for HCPs and patient support programs. Reliable real-world injection data, i.e. time and dose and additional parameters such as height and indications for treatment, provide personalized support to patients and caregivers across the treatment journey. Real-world data used in the clinic, include regular monitoring of adherence data and sharing these data with families during patients’ visits, so that a strategy between the HCP and family can be established to rectify poor adherence on individual case-based scenarios.

After applying the inclusion criteria, we first included 87 patients that used connected devices. This number was chosen based on a sample size calculation (assuming a small to medium effect size) and the feasibility given the total number of patients meeting the criteria at the clinic. All of the selected patients consented to the study. The connected device was the easypod™ drug delivery system which is an auto-injector for administration of recombinant growth hormone (r-hGH somatropin, Saizen^®^, Merck Healthcare KGaA, Darmstadt, Germany) (7). The device which is available in multiple languages includes a display that provides confirmation of the dose injected, last injection date and time and remaining dose in the cartridge (8). The information on date, time and dose allows adherence to be tracked and visualized via computer software, thus assisting HCPs in therapy decisions. The digital health software, known as the easypod™ clinical kit obtained the information from the device using a USB connection and a docking station in the clinic, which allowed for analysis on a connected computer with Microsoft Windows-based programs. Further modifications have been made to this digital health ecosystem from the easypod™ connect version 1, released in 2011 to the easypod™ connect Next system released in 2020 (9).

### 2.5 Non-connected patients

To select the patients with non-connected devices, a matched sampling technique was performed based on indication to balance the number of patients in both groups across indication.

### 2.6 Monitoring of hGH therapy: procedure at clinic when seeing connected and non-connected patients

When a patient using a connected device was seen, digital adherence data was examined and shared with the patient and family. In the case of suboptimal adherence, the patient was questioned non-threateningly with the aim of discovering the cause of the poor adherence and trying to solve this with the family. Connected patients were not seen more frequently and did not receive more attention or HCP support. Adjustment of the dose of hGH was similar in both groups. In the case of a patient on a non-connected device, recommendations regarding adherence were taken on the basis of what the family reported.

### 2.7 Data collection

We reviewed the following variables:

- Yearly patient visits during hGH therapy
- Treatment parameters: indication (GHD, SGA) and dose
- Demographic characteristics patients: age, sex (male, female)
- Device: Non-connected or connected
- Clinical parameters at start and at follow-up (first, second, third and fourth year of treatment): age (years), height, Puberty stage

Height was expressed as HSDS using the growth references of the 2010 Growth Studies of the Spanish population (10).

### 2.8 Statistical analysis

We assessed the normality of the data before the statistical analysis, and then proceeded to compare means and assess correlations. Multilevel linear regression analysis was performed with change in HSDS between treatment start and the first, second, third and fourth year of treatment as outcome, and the interaction between time and device (non-connected versus connected) as dependent variable. The analysis was performed both without adjustment (unadjusted effects) and with adjustment for age and HSDS at treatment, sex, indication, dose and Tanner stages during treatment, and time on treatment (adjusted effects). The analysis were performed in R version 4.4.0.

## 3 Results

In total, 174 patients, 98 females and 76 males, were included in the study. Treatment was administered with manual non-connected devices in 87 patients and 87 patients used connected devices. 57% of subjects had GHD and 43% were SGA, with a similar distribution in both groups. Sex and age at treatment start did not differ significantly between both groups. However, age was on average 0.9 years higher in the non-connected group (p<0.001) and therefore more patients reached higher Tanner stages at the 4y measurements (p=0.005). At start, the majority of patients were still prepuberal (85% and 74%). HSDS at treatment start was significantly higher (-2.6 vs -2.8 SDS, p<0.001) in the non-connected device group compared to the connected device group (**Table 1**).

**Table 1:** Background characteristics of the connected device and the non-connected device group.

Parameter	Category	Connected device group (N=87)	Non-connected device group (N=87)
Sex	Male (N, %)	36 (41%)	40 (46%)
	Female (N, %)	51 (59%)	47 (54%)
Indication	GHD (N, %)	50 (57%)	50 (57%)
	SGA (N, %)	37 (43%)	37 (43%)
Dose at treatment start for GHD	Median (min, max)	0.027 (0.024-0.034)	0.028 (0.024-0.030)
Dose at treatment start for SGA	Median (min, max)	0.035 (0.028-0.038)	0.035 (0.030-0.037)
Age at treatment start (in years)	Mean (SD)	6.6 (2.8)	7.5 (3.0)
HSDS at treatment start***	Mean (SD)	-2.8 (0.5)	-2.6 (0.4)
Tanner stage at treatment start	1 (N, %)	74 (85%)	64 (74%)
	2 (N, %)	11 (13%)	16 (18%)
	3 (N, %)	2 (2%)	7 (8%)
Tanner stage at 4 y measurement**	1 (N, %)	42 (48%)	44 (51%)
	2 (N, %)	13 (15%)	5 (6%)
	3 (N, %)	8 (9%)	4 (5%)
	4 (N, %)	19 (22%	14 (16%)
	5 (N, %)	5 (6%)	20 (23%)

**p<0.01, ***p<0.001.

### 3.1 Change in HSDS

In the connected device group, the change of HSDS, unadjusted for age and HSDS at treatment, sex, indication (SGA versus GHD), Tanner stage, and time on treatment, was significantly higher in the second (p=0.03), third (p=0.003) and fourth year (p<0.001) of treatment compared to the non-connected device group (**Table 2**). After four years, the change of HSDS was on average +0.28 higher in the connected versus the non-connected group.

**Table 2:** Linear regression analysis with change in HSDS as outcome.

Parameter Connected versus non- connected	B (SE)^1^	Adj. B (SE)^1,2^
Change in HSDS 1 y	0.12 (0.07)	0.09 (0.05)
Change in HSDS 2 y	0.16 (0.07)*	0.13 (0.07)*
Change in HSDS 3 y	0.22 (0.07)**	0.20 (0.07)**
Change in HSDS 4 y	0.28 (0.07)***	0.23 (0.07)***

^1^B is the coefficient or slope that represents the estimated difference in change in HSDS between the groups, the standard error of the coefficient (SE) measures the precision of B.

^2^Adjusted for age and HSDS at treatment, sex, indication, dose and Tanner stages during treatment, and time on treatment.

*p<0.05, **p<0.01, ***p<0.001.

**Figure 1:**
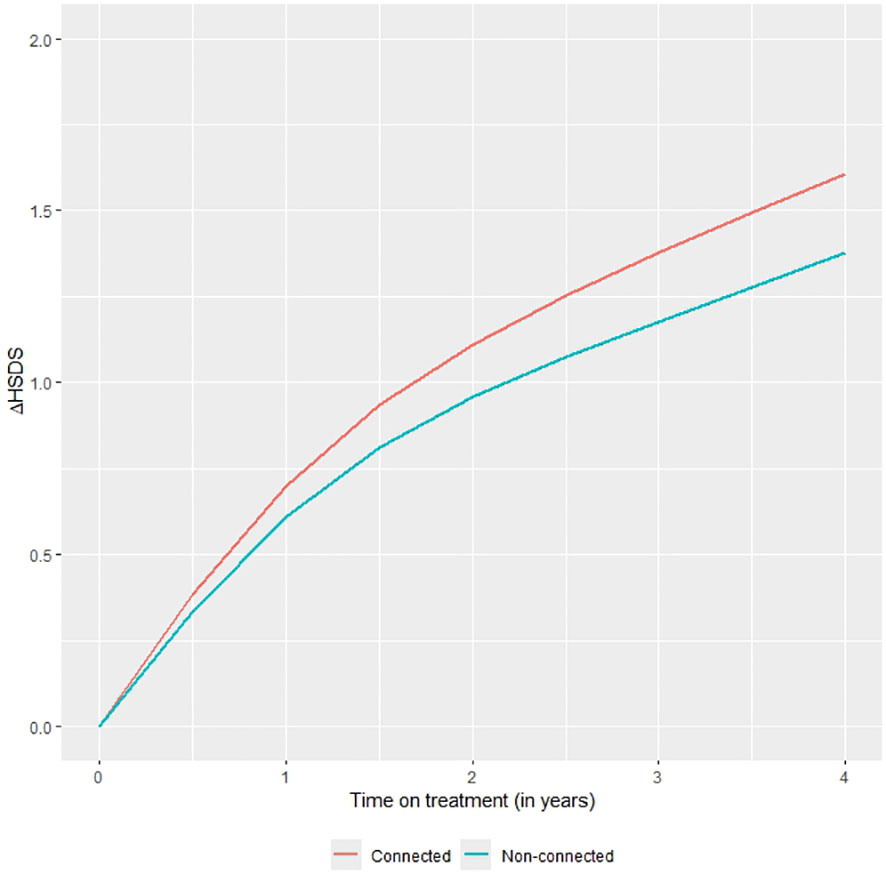
Mean change in HSDS by time on treatment in the connected device group and the non-connected device group with differences adjusted for baseline characteristics and other clinical parameters.

The change in HSDS, adjusted for the above variables, was also significantly higher in the second (p=0.048), third (p=0.004) and fourth (p<0.001) year of treatment in the connected device group compared to the non-connected device group. After four years, the change of HSDS was on average +0.23 higher in the connected versus the non-connected group. The reduction of this change after adjustment was mainly caused by differences in HSDS at treatment start between the two groups. **Figure 1** shows the adjusted change in HSDS over four years of treatment. Further stratification analysis showed that the effect of the connected device did not significantly differ by indication (p=0.63).

The difference in HSDS between the connected and non-connected device groups can be used to examine its impact on the final height of patients worldwide using WHO references (11, 12). If there is no further increase in the difference in HSDS after the fourth year of treatment, a +0.23 difference corresponds to an increase in adult height of 1.7 cm for males and 1.5 cm for females. If the same trend continues after the fourth year of treatment (on average 0.0575 SD/year), with a treatment duration of approximately 8.5 years in boys and 6.5 years in girls within GHD patients, this corresponds to an increase in adult height of 3.5 cm in GHD males and 2.4 cm in GHD females. Within the SGA group, with a treatment duration of approximately 9.5 y in boys and 8.0 y in girls, the corresponds to a difference of 4.0 cm in adult height in males and 3.0 cm in females.

## 4 Discussion

Our study found that the difference in change of HSDS between the connected device group and the manual non-connected device increased over time as they used hGH. Our study was not a randomized clinical trial. After four years of treatment, the change in height SDS was 0.23 higher in favor of the connected device group, after adjusting for potential confounders.

The finding of increased height gain during the first four years of hGH therapy in subjects treated with the connected device, compared to the non-connected device, is clinically relevant. Patients were not strictly randomized to the connected versus the non-connected group. There may therefore have been some bias in the selection of the groups, but a correction was made to adjust for potential confounders. The rates of adherence to hGH therapy were not formally assessed in this study. However, the degree of difference in change in HSDS between the two groups is significant.

The clinical journey of hGH therapy presents the child and family with a number of challenges over a period of many years. The burden of daily, or more recently weekly, subcutaneous injections imposes not only chronic and on-going discomfort, but restrictions of lifestyle and flexibility, normally enjoyed during childhood and adolescence. There is also the frustration of the child not seeing rapid fruits of success and benefit of the treatment because linear growth is a slow and steady process with increases in height only seen over months and years. Emphasis has correctly been made recently of the benefits of the child and family making their own choice of a number of hGH brands and their devices, rather than having a specific brand and injection device imposed on them. Patient choice is an important principle, not universally practiced, but directly linked to improved growth response to hGH (13).

Two key factors have been demonstrated to play a role in the short- and long-term growth response to hGH. These are first, the quality of adherence to the prescribed hGH regimen and secondly, persistence with the therapy, i.e. lack of discontinuation, as instructed (9). Non-adherence has been widely documented during pediatric hGH therapy, is linked to sub-optimal growth response (14) and has been shown to occur frequently (15). The reasons behind non-adherence are complex and include misconceptions about the consequences of missed doses, dissatisfaction with growth outcomes, a lack of understanding of the nature of the primary growth disorder and the rationale and evidence of benefit of hGH therapy and inadequate contact with a positive support from HCPs (16, 17).

The choice between an injection device, which records every injection and communicates the degree of hGH adherence both to the patient and to the HCP responsible for the treatment and a non-connected device from which knowledge of adherence is based on self-reported results is considerable. Rates of recorded adherence have been shown to be more accurate than self-reported results. The use of a connected device is consistent with the principles of individualized care by improving patient access to information, facilitating monitoring, intervention and communications with HCPs (18).

It could be argued that the families choosing the electronic device were more motivated and therefore more compliant. We do not believe this to be the case. All patients and families were equally and highly motivated. Also, we do not believe that the ‘electronic’ patients came from a higher social class. All degrees of social status were represented across the two groups. The patient receiving treatment using a connected device knows that adherence is being monitored and that the responsible HCP knows the real rate of adherence. This translates to increased effort by the patient to have better adherence and is known as the Hawthorne Effect (19). Over the years of treatment this effect will have an influence on growth response and overall height gain. Response to hGH therapy is highest in the first year of therapy and height velocity after initiation of treatment is directly linked to catch-up growth and the long-term height increase (20, 21).

As described above, an ecosystem is created with communication of data from the connected device to the patient and the HCP, which forms the basis of discussions regarding adherence, the growth response and any changes in therapy. Adherence has been shown to improve in these circumstances (22) and is particularly influenced by the individual psychological support a trained HCP is able to offer (23). This contrasts with the self-reported estimate of adherence offered by the patient treated with a non-connected device. Self-reported adherence data is inferior to formally recorded data (24).

In conclusion, the results reported in this article support the benefit of using the electronic connected device for treatment of pediatric growth disorders with hGH. Our study was not a randomized clinical trial, but a correction was made to adjust for potential confounders. The digital-human interface is of crucial importance to translate eHealth solutions into clear clinical benefits (25). Pediatric endocrinologists need to embrace the potential of digital tools and appropriately trained nursing and medical HCPs can in this way give personalized patient support which will contribute to improved patient care.

## Data Availability

The datasets for this article are not publicly available due to concerns regarding patient anonymity. Requests to access the datasets should be directed to Antonio de Arriba, adearriba@salud.aragon.es.
